# Supplementary material for: Adaptive communication between cell assemblies and “reader” neurons shapes flexible brain dynamics
Source: PLoS Biol. 2025 Dec 5;23(12):e3003505. doi: 10.1371/journal.pbio.3003505 (PMC12680171; doi:10.1371/journal.pbio.3003505)
Supplement: S1 Fig — (a) Left: spike trains of a subset of 35 simultaneously recorded units in prefrontal cortex during sleep (rasters: action potentials; gray ellipses surrounding colored ticks: co-activation events). Right: distribution of sizes of candidate prefrontal assemblies (number of members); proportion of prefrontal units that participated in candidate assemblies for each session (box and whiskers: distribution quartiles; dots: individual sessions); proportion of member spikes taking place in assembly activations for prefrontal members. (b) Same as (a) but for candidate amygdalar cell assemblies. (c) Z-scored cross-correlations between members of the same prefrontal (left) and amygdalar (right) assemblies, ordered by mode. (d) Same as in (c) for control pairs, illustrating that fewer pairs have modes at brief delays. (e) Averages of (c) (colored curves) and (d) (gray curves). Members of the same assemblies had significantly higher synchrony at short delays than control pairs (thick horizontal colored bars: p < 0.05, Monte–Carlo bootstraps). The data underlying this Figure can be found in https://doi.org/10.6080/K09W0CQP. (PDF) [file pbio.3003505.s001.pdf]

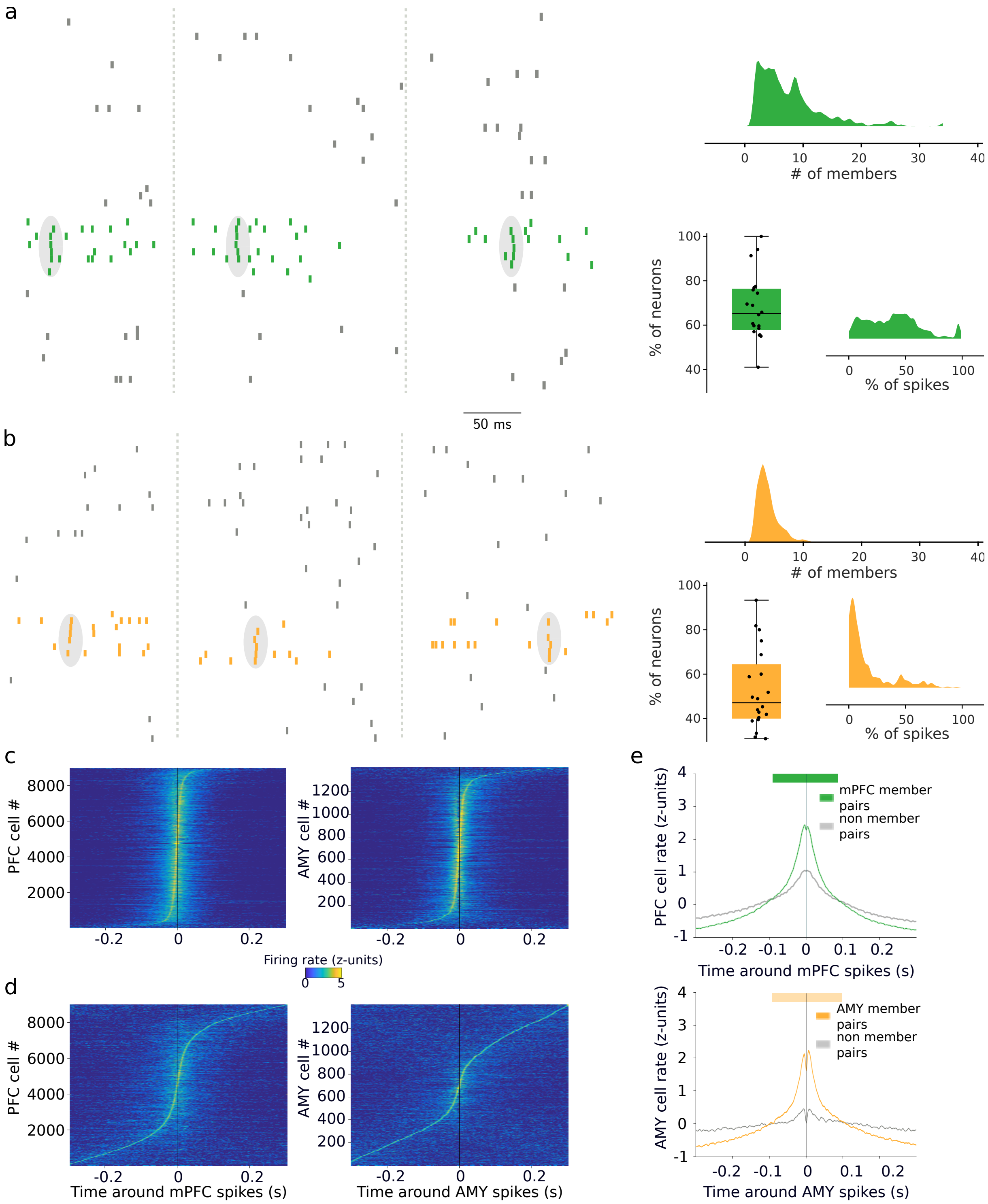

S1 Fig. Candidate cell assemblies in the cortico-amygdalar circuit. **a**, Left: spike trains of a subset of 35 simultaneously recorded units in prefrontal cortex during sleep (rasters: action potentials; gray ellipses surrounding colored ticks: co-activation events). Right: distribution of sizes of candidate prefrontal assemblies (number of members); proportion of prefrontal units that participated in candidate assemblies for each session (box and whiskers: distribution quartiles; dots: individual sessions); proportion of member spikes taking place in assembly activations for prefrontal members. **b**, Same as **a** but for candidate amygdalar cell assemblies. **c**, Z-scored cross-correlations between members of the same prefrontal (left) and amygdalar (right) assemblies, ordered by mode. **d**, Same as in (**c**) for control pairs, illustrating that fewer pairs have modes at brief delays. **e**, Averages of (**c**) (colored curves) and (**d**) (gray curves). Members of the same assemblies had significantly higher synchrony at short delays than control pairs (thick horizontal colored bars: $p < 0.05$, Monte-Carlo bootstraps). The data underlying this Figure can be found at CRCNS.
