# Supplementary material for: Adaptive communication between cell assemblies and “reader” neurons shapes flexible brain dynamics
Source: PLoS Biol. 2025 Dec 5;23(12):e3003505. doi: 10.1371/journal.pbio.3003505 (PMC12680171; doi:10.1371/journal.pbio.3003505)
Supplement: S2 Fig — (a) Number of candidate assemblies in the mPFC (left) and amygdala (right) in actual versus shuffled data preserving global rate fluctuations. (b) Proportion of candidate prefrontal (left) and amygdalar (right) assemblies that were independently detected in each half of the recorded data (***p < 0.001, Wilcoxon signed rank test). (c) Reader response score (see Materials and methods) for the first versus second halves of the recorded sessions. The data underlying this Figure can be found in https://doi.org/10.6080/K09W0CQP. (PDF) [file pbio.3003505.s002.pdf]

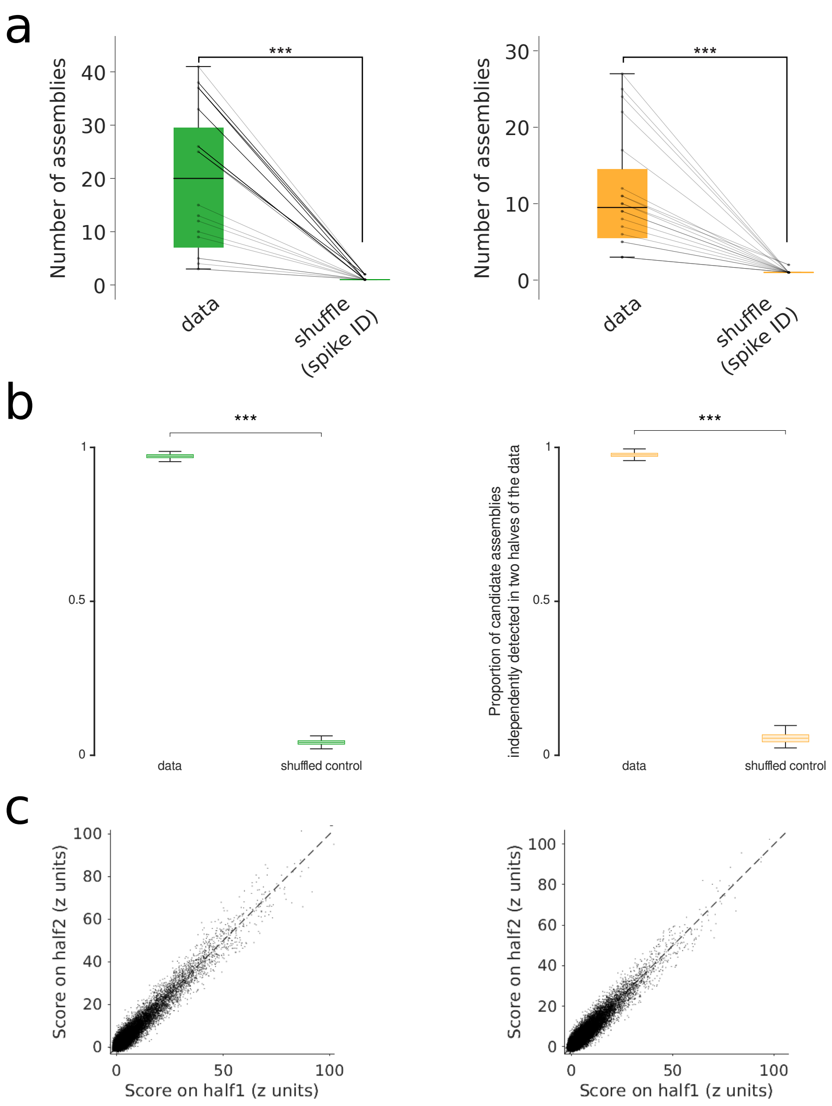

S2 Fig. Cross-validation of detection of candidate cell assemblies and readers. **a**, Number of candidate assemblies in the mPFC (left) and amygdala (right) in actual vs. shuffled data preserving global rate fluctuations. **b**, Proportion of candidate prefontal (left) and amygdalar (right) assemblies that were independently detected in each half of the recorded data (***$p < 0.001$, Wilcoxon signed rank test). **c**, Reader response score (see Methods) for the first vs second halves of the recorded sessions. The data underlying this Figure can be found at CRCNS.
