# Supplementary material for: Adaptive communication between cell assemblies and “reader” neurons shapes flexible brain dynamics
Source: PLoS Biol. 2025 Dec 5;23(12):e3003505. doi: 10.1371/journal.pbio.3003505 (PMC12680171; doi:10.1371/journal.pbio.3003505)
Supplement: S3 Fig — (a) Activations of a prefrontal assembly closely followed (10 ms–30 ms) by significant responses of an amygdalar neuron. Top: cell assembly weights (colored circles: assembly members, black circles: nonmembers). Bottom left: firing rate of an amygdalar neuron centered on all prefrontal assembly activations (mean ± s.e.m.). Thick orange horizontal bar indicates significant responses (p < 0.05: Monte–Carlo bootstrap test; see Materials and methods). Bottom right: example assembly activations (green curves: activation strength) followed by downstream spiking (rasters: prefrontal spikes within (green) or outside (gray) epochs of assembly activation; orange rasters: amygdalar spikes). Reader responses occurred ~20 ms after assembly activations. (b) Same as (a) for an amygdalar assembly and a downstream prefrontal neuron. The data underlying this Figure can be found in https://doi.org/10.6080/K09W0CQP. (PDF) [file pbio.3003505.s003.pdf]

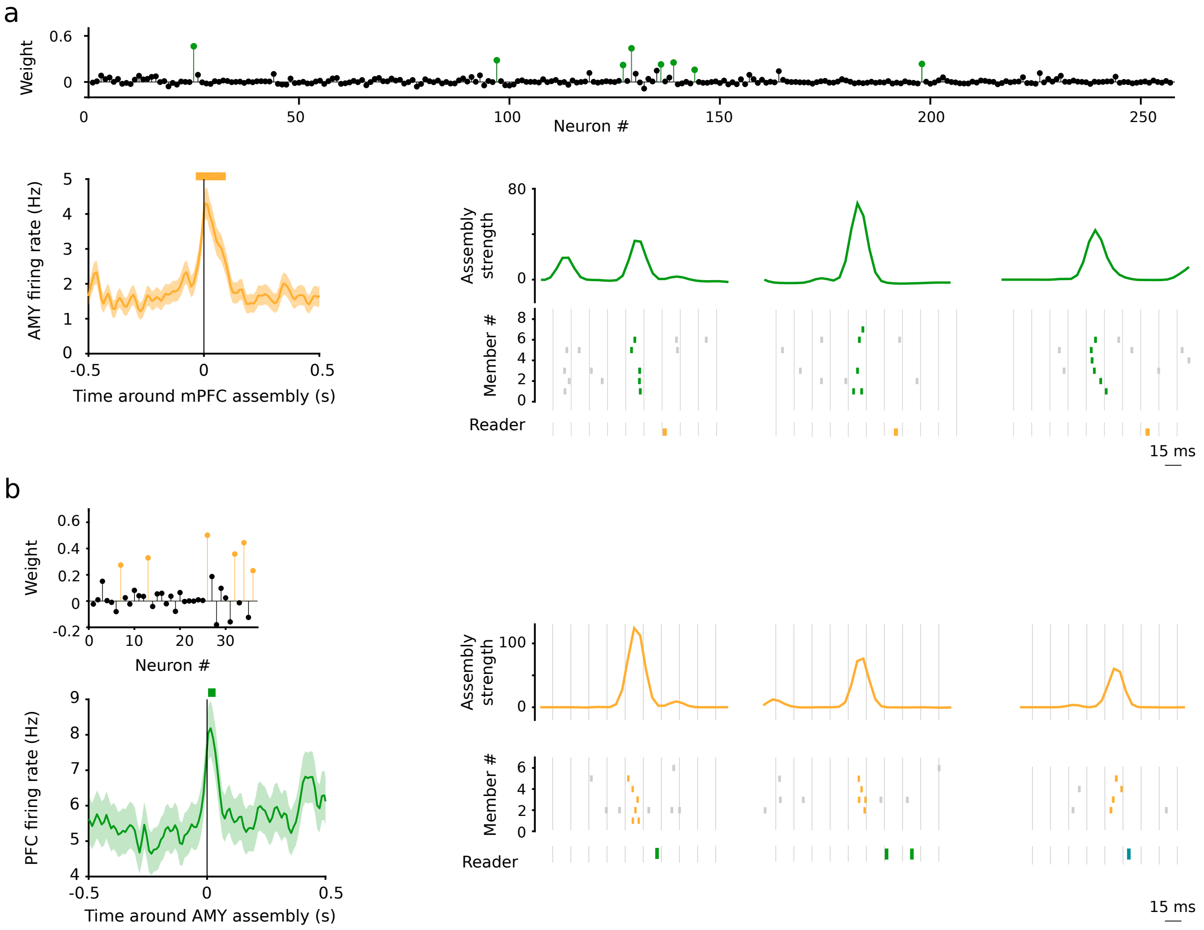

S3 Fig. Example assembly–reader pairs. **a**, Activations of a prefrontal assembly closely followed (10–30 ms) by significant responses of an amygdalar neuron. Top: cell assembly weights (colored circles: assembly members, black circles: non-members). Bottom left: firing rate of an amygdalar neuron centered on all prefrontal assembly activations (mean ± s.e.m.). Thick orange horizontal bar indicates significant responses ($p < 0.05$: Monte-Carlo bootstrap test; see Methods). Bottom right: example assembly activations (green curves: activation strength) followed by downstream spiking (rasters: prefrontal spikes within (green) or outside (gray) epochs of assembly activation; orange rasters: amygdalar spikes). Reader responses occurred ~20 ms after assembly activations. **b**, Same as (**a**) for an amygdalar assembly and a downstream prefrontal neuron. The data underlying this Figure can be found at CRCNS.
