# Supplementary material for: Adaptive communication between cell assemblies and “reader” neurons shapes flexible brain dynamics
Source: PLoS Biol. 2025 Dec 5;23(12):e3003505. doi: 10.1371/journal.pbio.3003505 (PMC12680171; doi:10.1371/journal.pbio.3003505)
Supplement: S4 Fig — (a) Percentage of significant pairs of candidate prefrontal assemblies and amygdalar readers as a function of the assembly–reader delay. Note that the number of significant pairs peaked for amygdala readers responding ~20 ms after candidate prefrontal assembly activation. (b) Same as (a) for candidate amygdalar assemblies and prefrontal readers. The data underlying this Figure can be found in https://doi.org/10.6080/K09W0CQP. (PDF) [file pbio.3003505.s004.pdf]

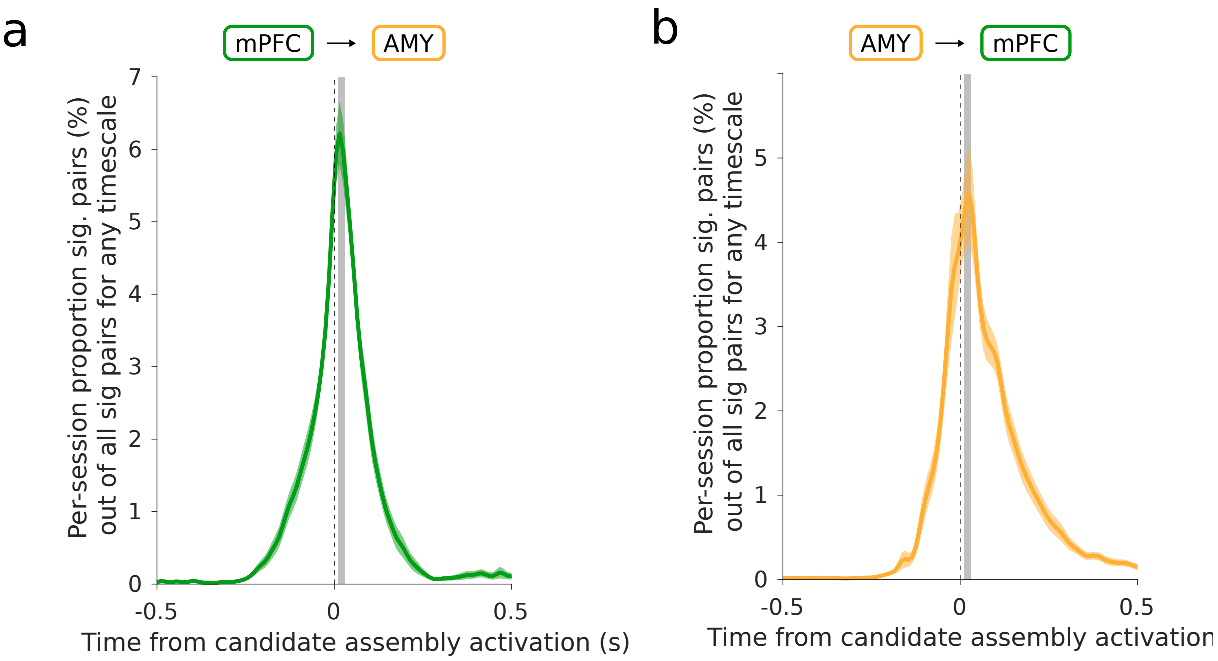

S4 Fig. Optimal delay for detecting assembly–reader pairs. **a**, Percentage of significant pairs of candidate prefrontal assemblies and amygdalar readers as a function of the assembly–reader delay. Note that the number of significant pairs peaked for amygdala readers responding ∼20 ms after candidate prefrontal assembly activation. **b**, Same as (**a**) for candidate amygdalar assemblies and prefrontal readers. The data underlying this Figure can be found at CRCNS.
