# Supplementary material for: Adaptive communication between cell assemblies and “reader” neurons shapes flexible brain dynamics
Source: PLoS Biol. 2025 Dec 5;23(12):e3003505. doi: 10.1371/journal.pbio.3003505 (PMC12680171; doi:10.1371/journal.pbio.3003505)
Supplement: S5 Fig — (a) Example amygdalar response to increasing numbers of simultaneously active prefrontal assembly members. Top left: Reader firing rate centered on assembly activation. Right: Reader firing rate for different numbers of coactive members. Bottom left: Superimposed response curves. (b) Same as (a), for example, prefrontal assembly and amygdalar reader. The data underlying this Figure can be found in https://doi.org/10.6080/K09W0CQP. (PDF) [file pbio.3003505.s005.pdf]

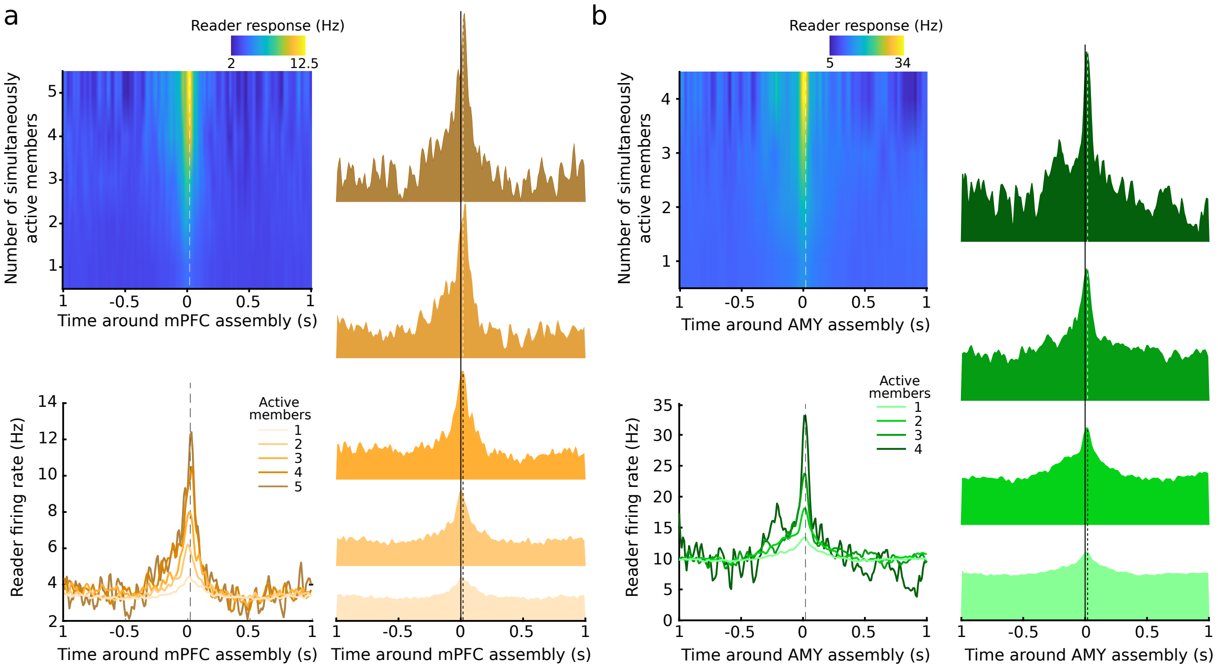

S5 Fig. Assembly members exert a synergistic influence on their targets: reader response rate increases with the number of co-active assembly members. **a**, Example amygdalar response to increasing numbers of simultaneously active prefrontal assembly members. Top left: Reader firing rate centered on assembly activation. Right: Reader firing rate for different numbers of co-active members. Bottom left: Superimposed response curves. **b**, Same as (**a**) for example prefrontal assembly and amygdalar reader. The data underlying this Figure can be found at CRCNS.
