# Supplementary material for: Adaptive communication between cell assemblies and “reader” neurons shapes flexible brain dynamics
Source: PLoS Biol. 2025 Dec 5;23(12):e3003505. doi: 10.1371/journal.pbio.3003505 (PMC12680171; doi:10.1371/journal.pbio.3003505)
Supplement: S6 Fig — (a) Average response of prefrontal readers to amygdalar assembly activations when the most effective members (i.e., the members whose spikes outside assembly activation epochs were followed by the largest response by the reader neuron at 10 ms–30 ms) of upstream assemblies were not recruited (leave 1-out, leave 2-out). (b) Same as (a) for amygdalar reader responses to member spikes of prefrontal assemblies. (PDF) [file pbio.3003505.s006.pdf]

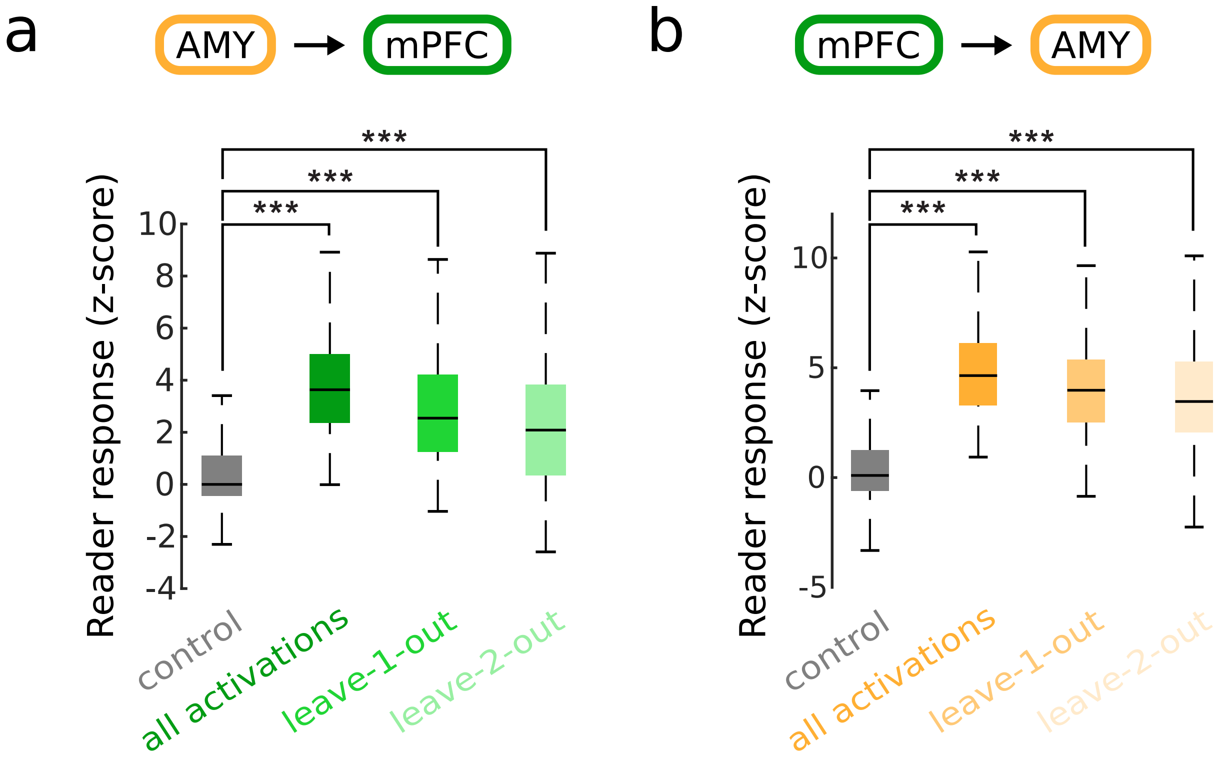

S6 Fig. Assembly members exert a synergistic influence on their targets: responses are not driven by single effective members. **a**, Average response of prefrontal readers to amygdalar assembly activations when the most effective members (i.e. the members whose spikes outside assembly activation epochs were followed by the largest response by the reader neuron at 10–30 ms) of upstream assemblies were not recruited (leave 1-out, leave 2-out). **b**, Same as (**a**) for amygdalar reader responses to member spikes of prefrontal assemblies. The data underlying this Figure can be found at CRCNS.
