# Supplementary material for: Adaptive communication between cell assemblies and “reader” neurons shapes flexible brain dynamics
Source: PLoS Biol. 2025 Dec 5;23(12):e3003505. doi: 10.1371/journal.pbio.3003505 (PMC12680171; doi:10.1371/journal.pbio.3003505)
Supplement: S7 Fig — (a) Top: Observed responses (colored curve: mean ± s.e.m.) of prefrontal readers compared to the estimated response of a linear reader (gray curve: mean ± s.e.m.). Inset: The observed response was greater than the linear estimate at 20 ms (***p < 0.001, Wilcoxon signed-rank test). Bottom: Supralinearity index of prefrontal reader responses. Dashed line: peak of reader responses to assembly activations at 20 ms. Inset: Supralinearity at 20 ms versus baseline (***p < 0.001, Wilcoxon signed-rank test). (b) Same as (a) for amygdalar reader responses to spikes of members of prefrontal assemblies. (c) Same as (a) for pooled responses of both amygdalar and prefrontal readers. (PDF) [file pbio.3003505.s007.pdf]

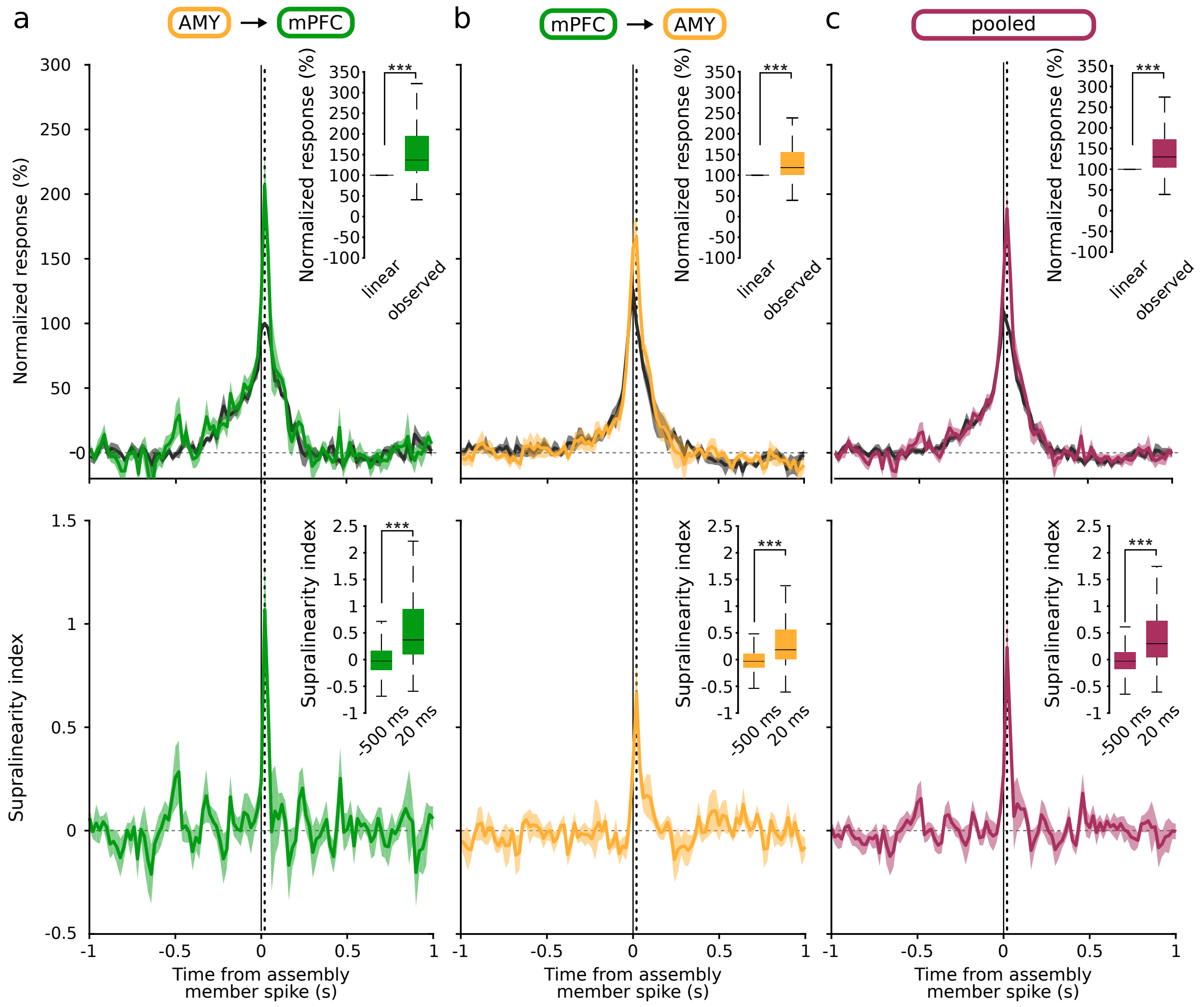

S7 Fig. Supralinearity of reader responses. **a**, Top: Observed responses (colored curve: mean ± s.e.m.) of prefrontal readers compared to the estimated response of a linear reader (gray curve: mean ± s.e.m.). Inset: The observed response was greater than the linear estimate at 20 ms (***$p < 0.001$, Wilcoxon signed-rank test). Bottom: Supralinearity index of prefrontal reader responses. Dashed line: peak of reader responses to assembly activations at 20 ms. Inset: Supralinearity at 20 ms vs baseline (***$p < 0.001$, Wilcoxon signed-rank test). **b**, Same as (**a**) for amygdalar reader responses to spikes of members of prefrontal assemblies. **c**, Same as (**a**) for pooled responses of both amygdalar and prefrontal readers. The data underlying this Figure can be found at `CRCNS`.
