# Supplementary material for: Adaptive communication between cell assemblies and “reader” neurons shapes flexible brain dynamics
Source: PLoS Biol. 2025 Dec 5;23(12):e3003505. doi: 10.1371/journal.pbio.3003505 (PMC12680171; doi:10.1371/journal.pbio.3003505)
Supplement: S8 Fig — (a) Response of prefrontal readers to amygdalar assembly members. Top: z-scored responses of reader neurons to two spikes emitted by different assembly members (AB, colored curve), compared to the control responses to two spikes emitted by the same assembly member (AA, gray curve) (mean ± sem). Bottom: Z-scored reader responses at 20 ms (***p < 0.001, Wilcoxon signed-rank test). (b) Same as (a) for amygdalar readers and PFC assembly members. (c) Same as (a) for pooled responses of both amygdalar and prefrontal readers. The data underlying this Figure can be found in https://doi.org/10.6080/K09W0CQP. (PDF) [file pbio.3003505.s008.pdf]

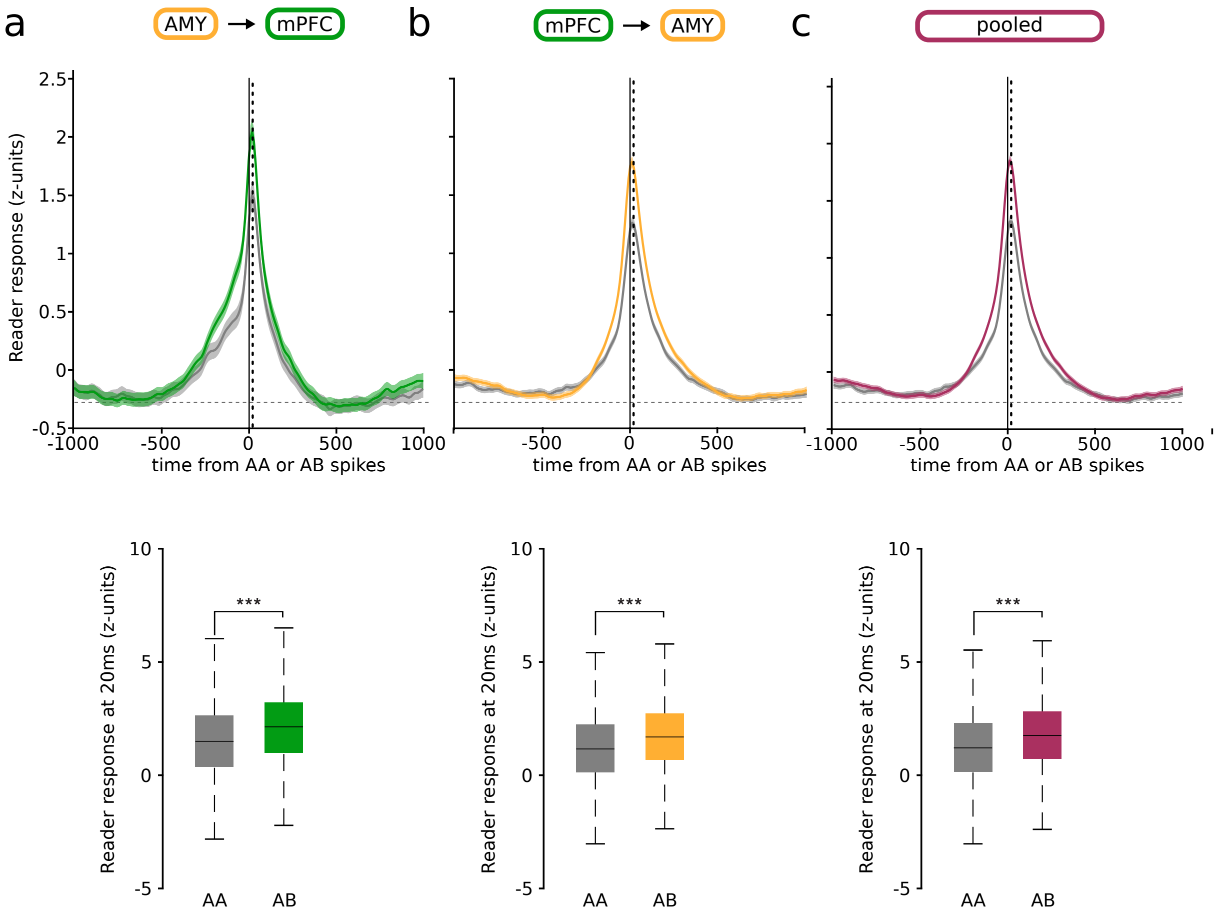

S8 Fig. The identity of participating members matters beyond their compound activity. **a**, Response of prefrontal readers to amygdalar assembly members. Top: z-scored responses of reader neurons to two spikes emitted by different assembly members (AB, colored curve), compared to the control responses to two spikes emitted by the same assembly member (AA, gray curve) (mean ± sem). Bottom: Z-scored reader responses at 20 ms (***$p < 0.001$, Wilcoxon signed-rank test). **b**, Same as (**a**) for amygdalar readers and PFC assembly members. **c**, Same as (**a**) for pooled responses of both amygdalar and prefrontal readers. The data underlying this Figure can be found at CRCNS.
