# Supplementary material for: Adaptive communication between cell assemblies and “reader” neurons shapes flexible brain dynamics
Source: PLoS Biol. 2025 Dec 5;23(12):e3003505. doi: 10.1371/journal.pbio.3003505 (PMC12680171; doi:10.1371/journal.pbio.3003505)
Supplement: S9 Fig — (a) Response of prefrontal readers to activations of amygdalar assemblies at varying time scales. Top and center: mean z-scored responses of a linear model versus the observed reader response, as a function of the time scale of the assembly. Bottom: difference between the two (observed response−linear estimate), for varying time scales. Thick colored horizontal bars indicate significant differences (p < 0.05, Monte–Carlo bootstrap test). (b) Same as (a) for amygdalar reader responses to member spikes of prefrontal assemblies. Note that in both cases, supralinearity is significantly greater than 0 for time scales up to 20 ms–25 ms. The data underlying this Figure can be found in https://doi.org/10.6080/K09W0CQP. (PDF) [file pbio.3003505.s009.pdf]

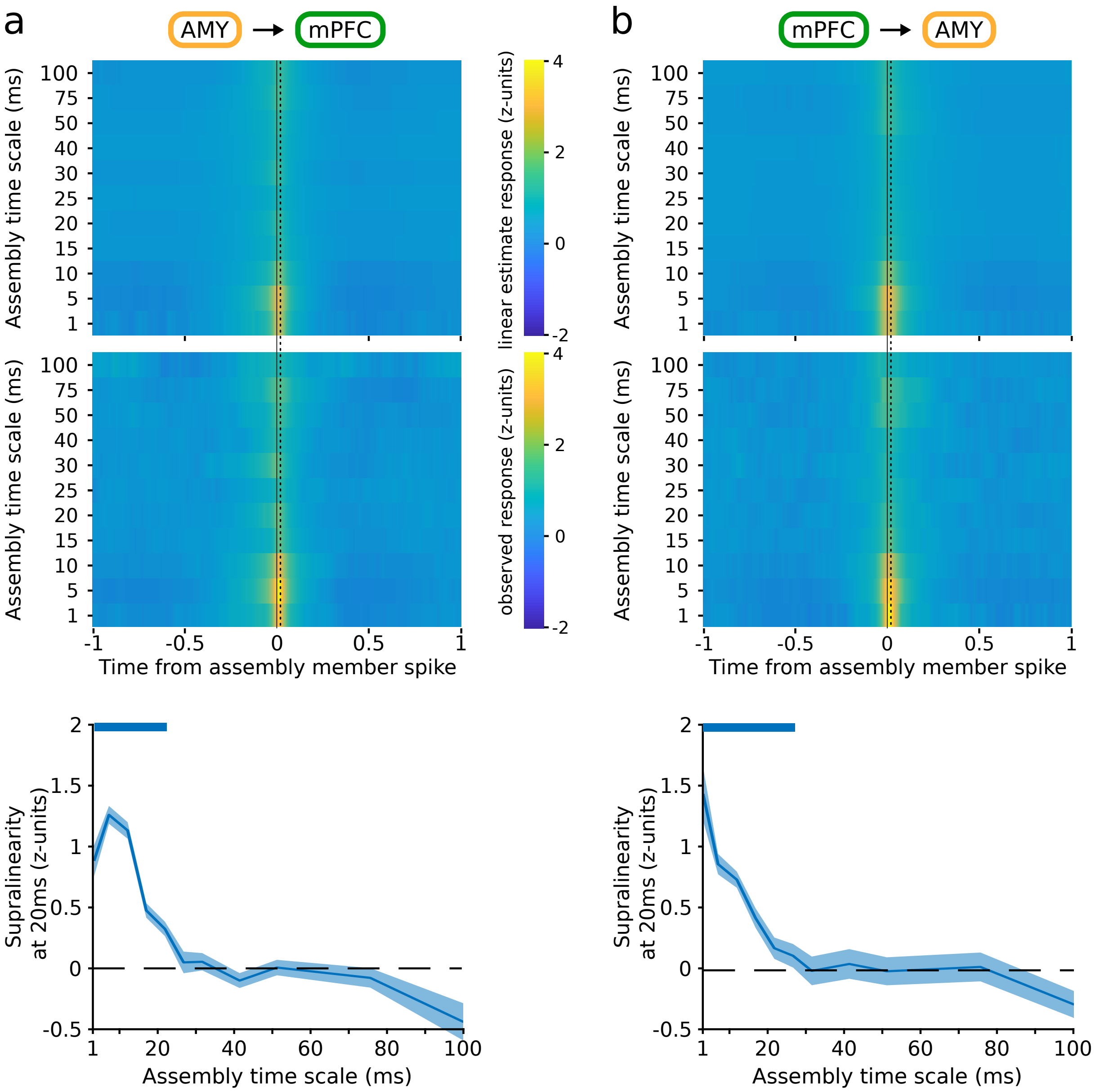

S9 Fig. Time scale of reader response supralinearity. **a**, Response of prefrontal readers to activations of amygdalar assemblies at varying time scales. Top and center: mean z-scored responses of a linear model vs the observed reader response, as a function of the time scale of the assembly. Bottom: difference between the two (observed response−linear estimate), for varying time scales. Thick colored horizontal bars indicate significant differences ($p < 0.05$, Monte-Carlo bootstrap test). **b**, Same as (**a**) for amygdalar reader responses to member spikes of prefrontal assemblies. Note that in both cases, supralinearity is significantly greater than 0 for time scales up to 20–25 ms. The data underlying this Figure can be found at CRCNS.
