# Supplementary material for: Adaptive communication between cell assemblies and “reader” neurons shapes flexible brain dynamics
Source: PLoS Biol. 2025 Dec 5;23(12):e3003505. doi: 10.1371/journal.pbio.3003505 (PMC12680171; doi:10.1371/journal.pbio.3003505)
Supplement: S10 Fig — (a) Response of prefrontal readers to two spikes emitted by different assembly members (AB), compared to the control responses to two spikes emitted by the same assembly member (AA), at varying time scales. Top and center: mean z-scored responses of reader neurons to spikes emitted by the same (AA, top) versus different (AB, center) members of an upstream assembly, as a function of the temporal delay between the two spikes. Bottom: difference between the two (AB−AA), for varying temporal delays. Thick colored horizontal bars indicate significant difference (p < 0.05, Monte–Carlo bootstrap test). (b) Same as (a) for amygdalar readers. The data underlying this Figure can be found in https://doi.org/10.6080/K09W0CQP. (PDF) [file pbio.3003505.s010.pdf]

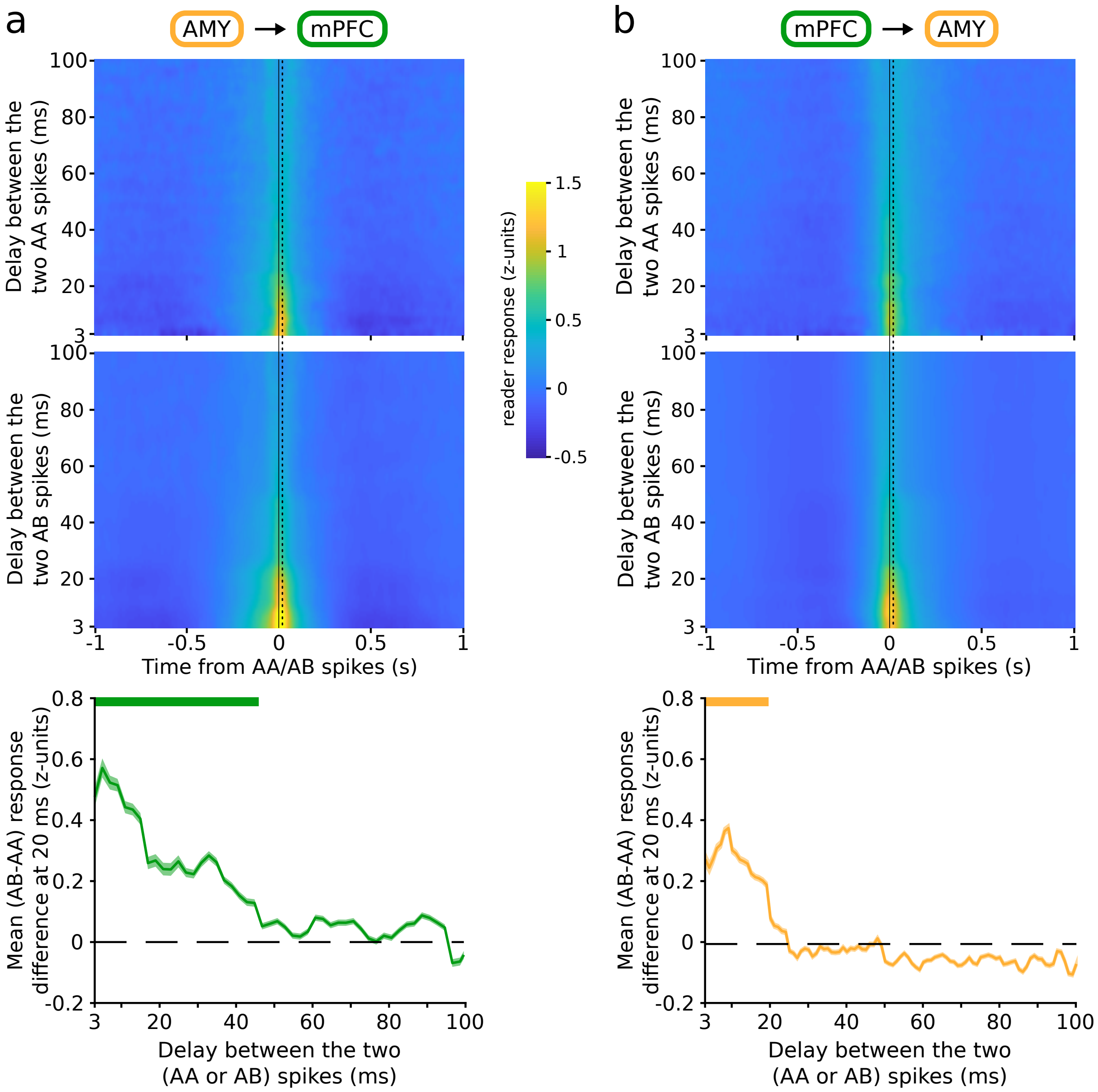

S10 Fig. Time scale of reader sensitivity to assembly member identity. a, Response of prefrontal readers to two spikes emitted by different assembly members (AB), compared to the control responses to two spikes emitted by the same assembly member (AA), at varying time scales. Top and center: mean z-scored responses of reader neurons to spikes emitted by the same (AA, top) vs different (AB, center) members of an upstream assembly, as a function of the temporal delay between the two spikes. Bottom: difference between the two (AB−AA), for varying temporal delays. Thick colored horizontal bars indicate significant difference ($p < 0.05$, Monte-Carlo bootstrap test). **b**, Same as (**a**) for amygdalar readers. The data underlying this Figure can be found at CRCNS.
