# Supplementary material for: Adaptive communication between cell assemblies and “reader” neurons shapes flexible brain dynamics
Source: PLoS Biol. 2025 Dec 5;23(12):e3003505. doi: 10.1371/journal.pbio.3003505 (PMC12680171; doi:10.1371/journal.pbio.3003505)
Supplement: S11 Fig — (a) Prefrontal reader responses to amygdalar assemblies. Top: Superimposed best-fit sigmoid curves of all assembly–reader pairs. Center: boost in reader response (relative to a proportional response) for all assembly–reader pairs as a function of the proportion of active assembly members. The gain was significant for the second and third quantiles (***p < 0.001, Wilcoxon signed-rank test), but not for the first quantile (*p < 0.05, Wilcoxon signed-rank test). Bottom: The data were better fit with sigmoidal than linear models (***p < 0.001, Wilcoxon signed-rank test). (b) Same as (a) for amygdalar reader responses to prefrontal assemblies. The data underlying this Figure can be found in https://doi.org/10.6080/K09W0CQP. (PDF) [file pbio.3003505.s011.pdf]

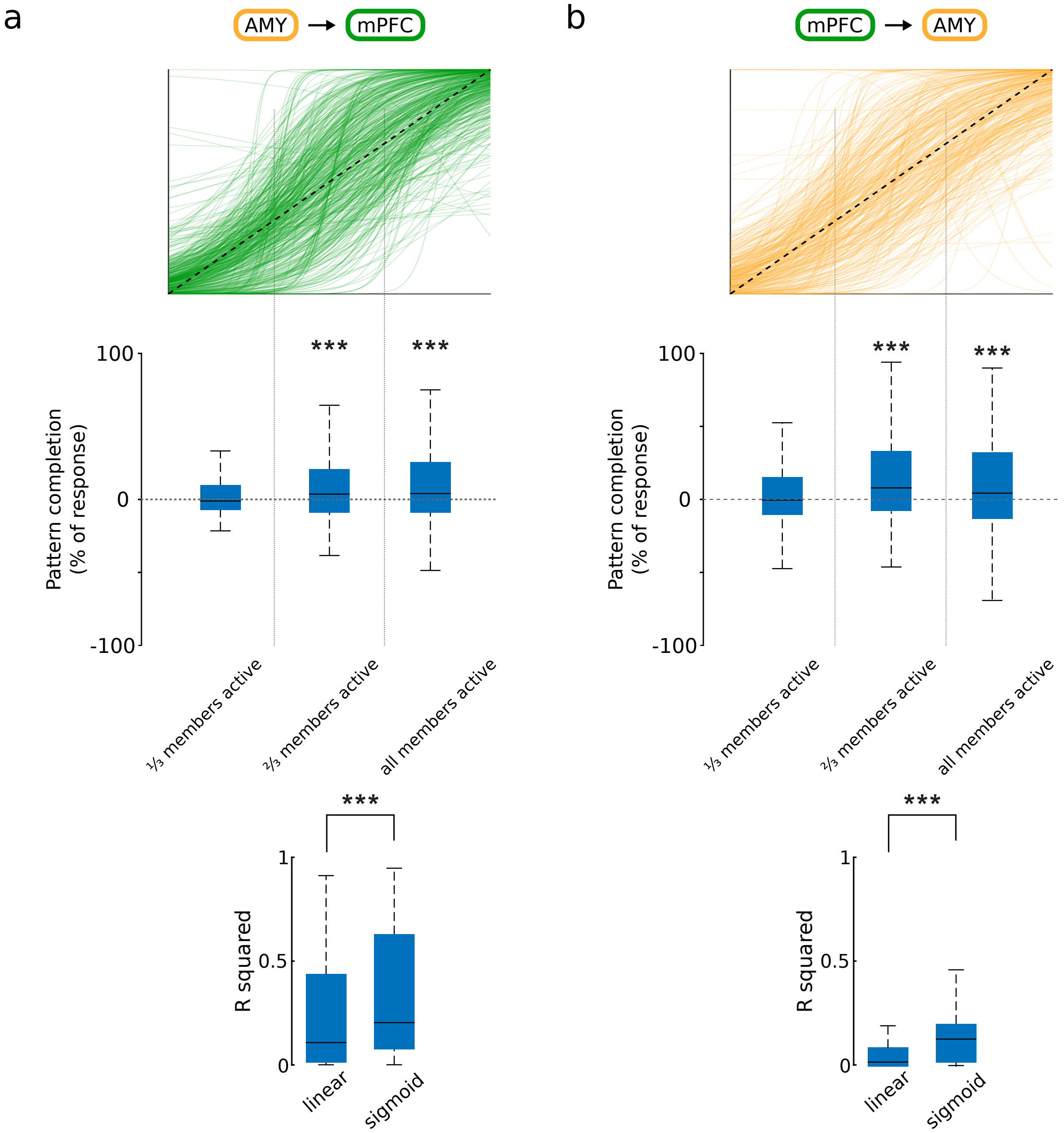

S11 Fig. The assembly–reader mechanism can implement pattern completion. **a**, Prefrontal reader responses to amygdalar assemblies. Top: Superimposed best-fit sigmoid curves of all assembly–reader pairs. Center: boost in reader response (relative to a proportional response) for all assembly–reader pairs as a function of the proportion of active assembly members. The gain was significant for the second and third quantiles (***$p < 0.001$, Wilcoxon signed-rank test), but not for the first quantile (*$p < 0.05$, Wilcoxon signed-rank test). Bottom: The data were better fit with sigmoidal than linear models (***$p < 0.001$, Wilcoxon signed-rank test). **b**, Same as (**a**) for amygdalar reader responses to prefrontal assemblies. The data underlying this Figure can be found at CRCNS.
