# Supplementary material for: Adaptive communication between cell assemblies and “reader” neurons shapes flexible brain dynamics
Source: PLoS Biol. 2025 Dec 5;23(12):e3003505. doi: 10.1371/journal.pbio.3003505 (PMC12680171; doi:10.1371/journal.pbio.3003505)
Supplement: S12 Fig — (a) Sparsity of prefrontal reader responses to amygdalar assemblies. Top left: Responses of an example prefrontal neuron to each amygdalar cell assembly in the recording session. Responses are selective for the paired assembly (dark green), compared to other assemblies (light green). Top right: Control responses of the same prefrontal neuron to surrogate assembly activations (shuffled assembly identities) are not selective. Center: distribution of sparsity for nonreader (left) and reader (right) neurons, compared to control sparsity computed from shuffled data (gray). Note that the observed responses are sparser than the shuffled control (***p < 0.001, Wilcoxon signed rank test). Bottom: Sparsity increase from shuffle, for reader versus nonreader neurons (***p < 0.001, Wilcoxon rank sum test). (b) Same as (a) for amygdalar reader responses to prefrontal assemblies. The data underlying this Figure can be found in https://doi.org/10.6080/K09W0CQP. (PDF) [file pbio.3003505.s012.pdf]

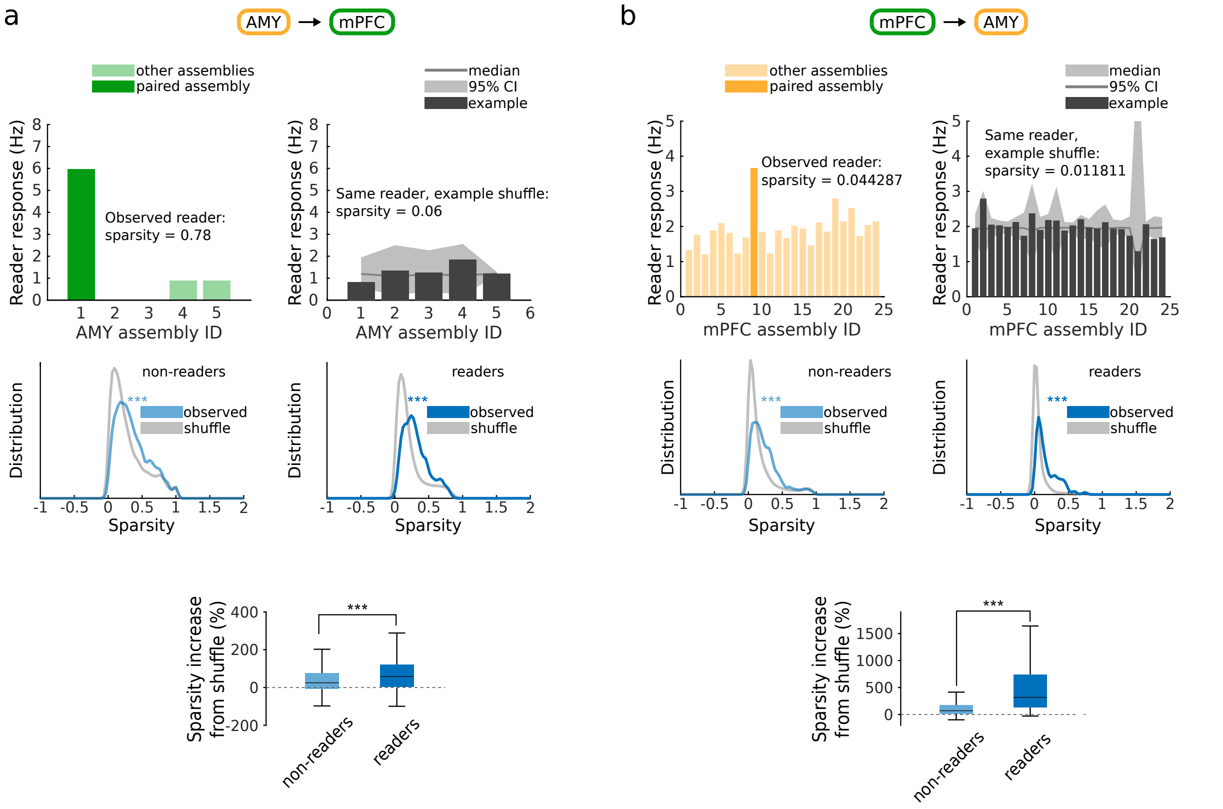


**S12 Fig. The assembly–reader mechanism can implement pattern separation: reader responses are selective for specific assemblies.** **a**, Sparsity of prefrontal reader responses to amygdalar assemblies. Top left: Responses of an example prefrontal neuron to each amygdalar cell assembly in the recording session. Responses are selective for the paired assembly (dark green), compared to other assemblies (light green). Top right: Control responses of the same prefrontal neuron to surrogate assembly activations (shuffled assembly identities) are not selective. Center: distribution of sparsity for non-reader (left) and reader (right) neurons, compared to control sparsity computed from shuffled data (gray). Note that the observed responses are sparser than the shuffled control (***$p < 0.001$, Wilcoxon signed rank test). Bottom: Sparsity increase from shuffle, for reader vs non-reader neurons (***$p < 0.001$, Wilcoxon rank sum test). **b**, Same as (**a**) for amygdalar reader responses to prefrontal assemblies. The data underlying this Figure can be found at CRCNS.
