# Supplementary material for: Adaptive communication between cell assemblies and “reader” neurons shapes flexible brain dynamics
Source: PLoS Biol. 2025 Dec 5;23(12):e3003505. doi: 10.1371/journal.pbio.3003505 (PMC12680171; doi:10.1371/journal.pbio.3003505)
Supplement: S13 Fig — (a) Pattern separation in prefrontal reader responses to amygdalar assemblies. Top: prefrontal reader responses to activation of a paired assembly (left) versus a different but overlapping (≥25%) assembly, sorted by discrimination index. Responses above the white dotted line manifested significant pattern separation (greater discrimination indices than shuffled data, p < 0.05, Wilcoxon rank sum test). Bottom: Discrimination indices for overlapping assemblies (x-axis: number of overlapping members) were greater for observed than shuffled data (***p < 0.001, Wilcoxon rank sum test). (b) Same as (a) for amygdalar reader responses to prefrontal assemblies. The data underlying this Figure can be found in https://doi.org/10.6080/K09W0CQP. (PDF) [file pbio.3003505.s013.pdf]

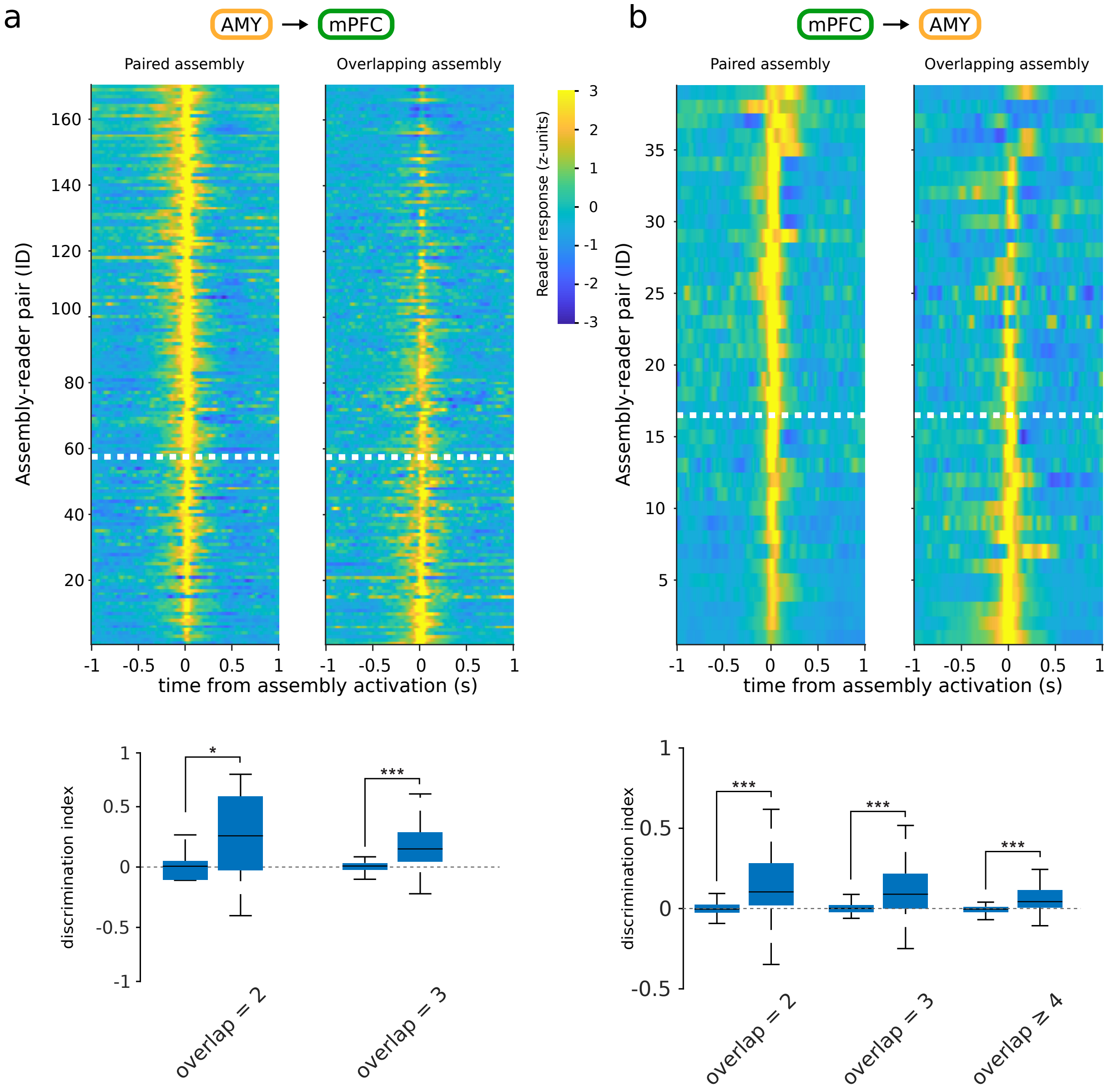

S13 Fig. The assembly–reader mechanism can implement pattern separation: readers can discriminate between overlapping assemblies. **a**, Pattern separation in prefrontal reader responses to amygdalar assemblies. Top: prefrontal reader responses to activation of a paired assembly (left) vs a different but overlapping ($\geq 25\%$) assembly, sorted by discrimination index. Responses above the white dotted line manifested significant pattern separation (greater discrimination indices than shuffled data, $p < 0.05$, Wilcoxon rank sum test). Bottom: Discrimination indices for overlapping assemblies (x-axis: number of overlapping members) were greater for observed than shuffled data (***$p < 0.001$, Wilcoxon rank sum test). **b**, Same as (**a**) for amygdalar reader responses to prefrontal assemblies. The data underlying this Figure can be found at CRCNS.
