# Supplementary material for: Adaptive communication between cell assemblies and “reader” neurons shapes flexible brain dynamics
Source: PLoS Biol. 2025 Dec 5;23(12):e3003505. doi: 10.1371/journal.pbio.3003505 (PMC12680171; doi:10.1371/journal.pbio.3003505)
Supplement: S14 Fig — (a) Cell assembly weights of three representative prefrontal assemblies (colored circles: assembly members, black circles: nonmembers), corresponding to eigenvalues 1, 28, and 38. Whereas all members of assemblies 1 and 28 were of the same (positive) sign, assembly 38 included members with both positive and negative weights (“mixed-sign assembly”). (b) The separation between members and nonmembers was significantly better in same-sign assemblies than mixed-sign assemblies (***p < 0.001, Wilcoxon rank sum test). (c) Mixed-sign assemblies had significantly more members than same-sign assemblies (***p < 0.001, Wilcoxon rank sum test). The data underlying this Figure can be found in https://doi.org/10.6080/K09W0CQP. (PDF) [file pbio.3003505.s014.pdf]

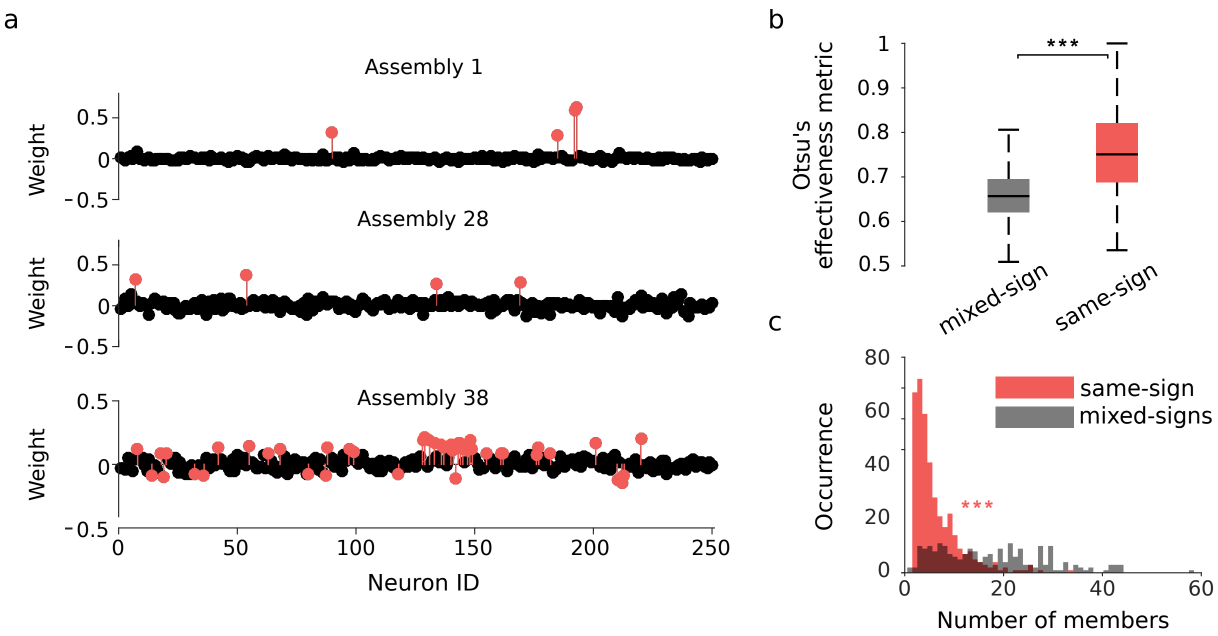

S14 Fig. Selection of candidate cell assemblies with same-sign component weights. **a**, Cell assembly weights of three representative prefrontal assemblies (colored circles: assembly members, black circles: non-members), corresponding to eigenvalues 1, 28 and 38. Whereas all members of assemblies 1 and 28 were of the same (positive) sign, assembly 38 included members with both positive and negative weights ('mixed-sign assembly'). **b**, The separation between members and non-members was significantly better in same-sign assemblies than mixed-sign assemblies (***$p < 0.001$, Wilcoxon rank sum test). **c**, Mixed-sign assemblies had significantly more members than same-sign assemblies (***$p < 0.001$, Wilcoxon rank sum test). The data underlying this Figure can be found at CRCNS.
