# Supplementary material for: Adaptive communication between cell assemblies and “reader” neurons shapes flexible brain dynamics
Source: PLoS Biol. 2025 Dec 5;23(12):e3003505. doi: 10.1371/journal.pbio.3003505 (PMC12680171; doi:10.1371/journal.pbio.3003505)
Supplement: S15 Fig — (a) An example assembly recorded in the prefrontal cortex. Red dots: assembly members. (b) Example activation of assembly shown in (a). Top: Assembly activation strength computed using either the activity of all cells (gray curve) or the activity of member cells only (red curve). Bottom: Raster plot of the activity of a representative subset of neurons, ordered by absolute weight (vertical ticks: action potentials; red ticks: member cells; gray ticks: nonmember cells; shaded rectangle: putative assembly activation). All three members were active, resulting in a high activation strength in both curves. (c) Same as (b) but for an instance in which only a single assembly member was active, at the same time as two nonmembers. The corresponding peak in the gray curve would result in incorrect detection of an activation of the assembly. This spurious peak is absent from the red curve, where activity strength is computed using only assembly members. (d) Left: Proportion of assembly members coactive around peaks in the assembly activation strength computed using the activity of member cells only (red) or using the activity of all cells (gray). Right: using the activity of member cells results in detection of assembly activation events with greater proportions of coactive members (*** p < 0.001, Wilcoxon signed rank test). The data underlying this Figure can be found in https://doi.org/10.6080/K09W0CQP. (PDF) [file pbio.3003505.s015.pdf]

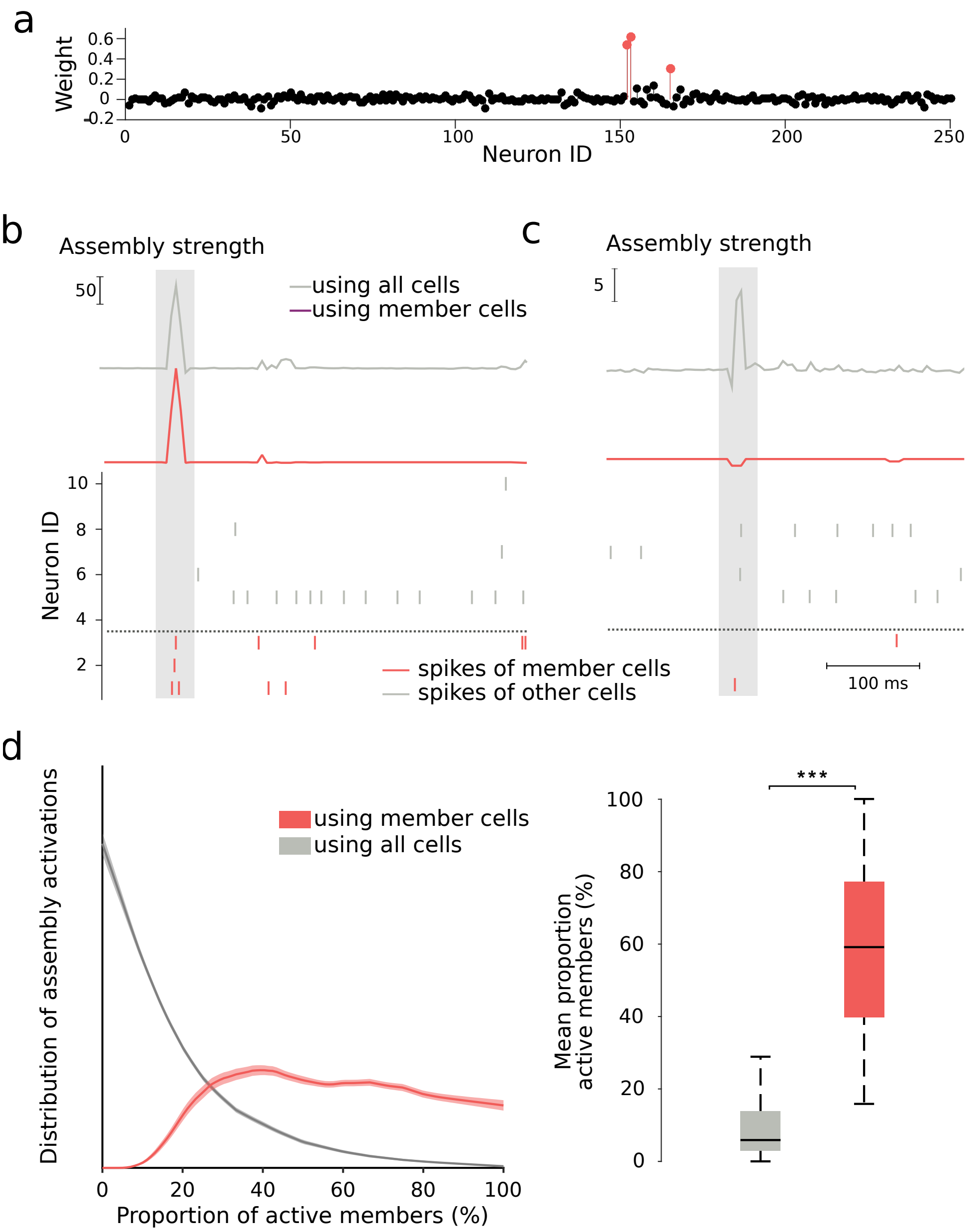

S15 Fig. Computation of assembly activation strength. **a**, An example assembly recorded in the prefrontal cortex. Red dots: assembly members. **b**, Example activation of assembly shown in (**a**). Top: Assembly activation strength computed using either the activity of all cells (gray curve) or the activity of member cells only (red curve). Bottom: Raster plot of the activity of a representative subset of neurons, ordered by absolute weight (vertical ticks: action potentials; red ticks: member cells; gray ticks: non-member cells; shaded rectangle: putative assembly activation). All three members were active, resulting in a high activation strength in both curves. **c**, Same as (**b**) but for an instance in which only a single assembly member was active, at the same time as two non-members. The corresponding peak in the gray curve would result in incorrect detection of an activation of the assembly. This spurious peak is absent from the red curve, where activity strength is computed using only assembly members. **d**, Left: Proportion of assembly members co-active around peaks in the assembly activation strength computed using the activity of member cells only (red) or using the activity of all cells (gray). Right: using the activity of member cells results in detection of assembly activation events with greater proportions of co-active members (***$p < 0.001$, Wilcoxon signed rank test)). The data underlying this Figure can be found at CRCNS.
