# Supplementary material for: Adaptive communication between cell assemblies and “reader” neurons shapes flexible brain dynamics
Source: PLoS Biol. 2025 Dec 5;23(12):e3003505. doi: 10.1371/journal.pbio.3003505 (PMC12680171; doi:10.1371/journal.pbio.3003505)
Supplement: S1 Table — (PDF) [file pbio.3003505.s016.pdf]

	Overlapping members					
	**0**	**1**	**2**	**3**	**4**	**5**
Animal 1, session 1	14/15	1/15	0/15	0/15	0/15	0/15
Animal 1, session 2	39/45	4/45	1/45	0/45	0/45	1/45
Animal 1, session 3	19/21	0/21	0/21	1/21	1/21	0/21
Animal 1, session 4	19/21	0/21	2/21	0/21	0/21	0/21
Animal 1, session 5	9/10	1/10	0/10	0/10	0/10	0/10
Animal 2, session 1	168/190	15/190	6/190	1/190	0/190	0/190
Animal 2, session 2	221/253	23/253	7/253	2/253	0/253	0/253
Animal 2, session 3	234/276	30/276	9/276	2/276	1/276	0/276
Animal 2, session 4	174/210	25/210	6/210	5/210	0/210	0/210
Animal 2, session 5	184/210	17/210	7/210	1/210	1/210	0/210
Animal 3, session 1	116/120	2/120	2/120	0/120	0/120	0/120
Animal 3, session 2	122/136	9/136	3/136	2/136	0/136	0/136
Animal 3, session 3	137/153	12/153	2/153	1/153	1/153	0/153
Animal 3, session 4	127/136	8/136	0/136	1/136	0/136	0/136
Animal 3, session 5	126/136	7/136	3/136	0/136	0/136	0/136
Animal 4, session 1	3/3	0/3	0/3	0/3	0/3	0/3
Animal 4, session 2	9/10	1/10	0/10	0/10	0/10	0/10
Animal 4, session 3	1/1	0/1	0/1	0/1	0/1	0/1
Animal 4, session 4	1/3	1/3	1/3	0/3	0/3	0/3
Animal 4, session 5	7/10	2/10	1/10	0/10	0/10	0/10

S1 Table: Overlapping members in candidate prefrontal assemblies by animal and session (fraction m/n in column c indicates that m pairs of candidate assemblies out of n had c overlapping members).
