# Supplementary material for: Adaptive communication between cell assemblies and “reader” neurons shapes flexible brain dynamics
Source: PLoS Biol. 2025 Dec 5;23(12):e3003505. doi: 10.1371/journal.pbio.3003505 (PMC12680171; doi:10.1371/journal.pbio.3003505)
Supplement: S2 Table — (PDF) [file pbio.3003505.s017.pdf]

	Overlapping members			
	**0**	**1**	**2**	**3**
Animal 1, session 1	3/3	0/3	0/3	0/3
Animal 1, session 2	15/15	0/15	0/15	0/15
Animal 1, session 3	35/36	1/36	0/36	0/36
Animal 1, session 4	53/55	1/55	1/55	0/55
Animal 1, session 5	15/15	0/15	0/15	0/15
Animal 2, session 1	144/153	7/153	1/153	1/153
Animal 2, session 2	77/78	1/78	0/78	0/78
Animal 2, session 3	130/136	6/136	0/136	0/136
Animal 2, session 4	49/55	6/55	0/55	0/55
Animal 2, session 5	69/78	8/78	1/78	0/78
Animal 3, session 1	6/6	0/6	0/6	0/6
Animal 3, session 2	8/10	2/10	0/10	0/10
Animal 3, session 3	7/10	3/10	0/10	0/10
Animal 3, session 4	6/6	0/6	0/6	0/6
Animal 3, session 5	9/10	1/10	0/10	0/10
Animal 4, session 1	1/3	2/3	0/3	0/3
Animal 4, session 2	6/6	0/6	0/6	0/6
Animal 4, session 3	6/6	0/6	0/6	0/6
Animal 4, session 2	5/6	1/6	0/6	0/6
Animal 4, session 5	1/1	0/1	0/1	0/1

S2 Table: Overlapping members in candidate amygdalar assemblies by animal and session (fraction m/n in column c indicates that m pairs of candidate assemblies out of n had c overlapping members).
